# Deep sequencing of a recurrent oligodendroglioma and the derived xenografts reveals new insights into the evolution of human oligodendroglioma and candidate driver genes

**DOI:** 10.18632/oncotarget.26950

**Published:** 2019-06-04

**Authors:** Nadin D. Exner, Jaime Alberto Campos Valenzuela, Khalil Abou-El-Ardat, Hrvoje Miletic, Peter C. Huszthy, Petra M. Radehaus, Evelin Schröck, Rolf Bjerkvig, Lars Kaderali, Barbara Klink, Janice M. Nigro

**Affiliations:** ^1^Institut für Klinische Genetik, Medizinische Fakultät Carl Gustav Carus, Technische Universität Dresden, Dresden, Germany; ^2^University of Applied Sciences Mittweida, Department of Applied Informatics & Biosciences, Mittweida, Germany; ^3^Institut für Medizinische Informatik und Biometrie, Medizinische Fakultät Carl Gustav Carus, Technische Universität Dresden, Dresden, Germany; ^4^Department of Pathology, Haukeland University Hospital, Bergen, Norway; ^5^Department of Biomedicine, University of Bergen, Bergen, Norway; ^6^Oslo University Hospital-Rikshospitalet, Department of Immunology, Oslo, Norway; ^7^German Cancer Consortium (DKTK), Dresden, Germany; ^8^German Cancer Research Center (DKFZ), Heidelberg, Germany; ^9^Center for Molecular Tumor Diagnostics, National Center for Tumor Diseases (NCT), Dresden, Germany; ^10^Oncology Department, Luxembourg Institute of Health, Val Fleuri, Luxembourg; ^11^University Medicine Greifswald, Institute of Bioinformatics, Greifswald, Germany; ^12^Centre national de Génétique, Laboratoire National de Santé, Dudelange, Luxembourg; ^*^Co-senior authors

**Keywords:** exome sequencing, IDH1, oligodendroglioma, SNV, xenograft

## Abstract

We previously reported the establishment of a rare xenograft derived from a recurrent oligodendroglioma with 1p/19q codeletion. Here, we analyzed in detail the exome sequencing datasets from the recurrent oligodendroglioma (WHO grade III, recurrent O^2010^) and the first-generation xenograft (xenograft^1^). Somatic SNVs and small InDels (*n* = 80) with potential effects at the protein level in recurrent O^2010^ included variants in *IDH1* (NM_005896:c.395G>A; p. Arg132His), *FUBP1* (NM_003902:c.1307_1310delTAGA; p.Ile436fs), and *CIC* (NM_015125:c.4421T>G; p.Val1474Gly). All but 2 of these 80 variants were also present in xenograft^1^, along with 7 new variants. Deep sequencing of the 87 SNVs and InDels in the original tumor (WHO grade III, primary O^2005^) and in a second-generation xenograft (xenograft^2^) revealed that only 11 variants, including *IDH1* (NM_005896:c.395G>A; p. Arg132His), *PSKH1* (NM_006742.2:c.650G>A; p.Arg217Gln), and *SNX12* (NM_001256188:c.470G>A; p.Arg157His), along with a variant in the *TERT* promoter (C250T, NM_198253.2: c.-146G>A), were already present in primary O^2005^. Allele frequencies of the 11 variants were calculated to assess their potential as putative driver genes. A missense change in *NDST4* (NM_022569:c.2392C>G; p.Leu798Val) on 4q exhibited an increasing allele frequency (~ 20%, primary O^2005^, 80%, recurrent O^2010^ and 100%, xenograft^1^), consistent with a selection event. Sequencing of *NDST4* in a cohort of 15 oligodendrogliomas, however, revealed no additional cases with potential protein disrupting variants. Our analysis illuminated a tumor evolutionary series of events, which included 1p/19q codeletion, *IDH1* R132H, and *TERT* C250T as early events, followed by loss of function of *NDST4* and mutations in *FUBP1* and *CIC* as late events.

## INTRODUCTION

Few of the extensive number of mutations compiled for human tumors, since the advent of exome sequencing, have impacted our understanding of tumor biology like those in human diffuse low grade gliomas. These tumors represent a unique cancer subgroup largely because of a distinctive subset of variants, which includes isocitrate dehydrogenase 1/2 (*IDH1/IDH2*) [1–3]. These variants create a neomorphic enzyme that generates a metabolite, 2-hydroxyglutarate (2-HG), not normally detectable in the human brain [4]. The metabolite disrupts function of proteins involved in epigenetic modification and interrupts the differentiation process [5, 6]. Examination of other tumor types has revealed a role for *IDH1/2* variants in the development of these cancers as well [7–9]. Yet, there is a clear need to further delineate the significance of these mutations with regard to the biology of tumors in general and particularly in the clinic.

Despite a less aggressive clinical course than other malignant gliomas, such as glioblastoma, the diffuse glioma subtype, oligodendroglioma, remains an incurable disease with a range of survival times after the primary diagnosis. Oligodendroglioma exhibits a high frequency of *IDH1/2* variants, particularly in cases harboring the signature genetic event, 1p/19q codeletion [3]. The putative targets of 1p/19q codeletion, tumor suppressor genes *FUBP1* and *CIC*, along with *TERT* promoter and *IDH1/2* variants, generate a unique constellation of genetic aberrations which distinguish these tumors not only from other gliomas, but from most other human cancers [10–12]. However, our understanding of the biology of these tumors and the molecular mechanisms at play during therapy are extremely limited due until recently to the lack of appropriate models that reflect the human disease. Previously, we orthotopically engrafted a human oligodendroglioma in two successive implantations [13]. Other *in vivo* models of oligodendroglioma have been described, but only recently has a more detailed analysis of the underlying genetic alterations been reported [14–17].

In this work, a complete analysis of the exome sequence dataset from a recurrent primary oligodendroglioma (recurrent O^2010^) and the derived xenograft (xenograft^1^) was performed. The single nucleotide variants (SNVs) and insertions/deletions (InDels) detected were subsequently analyzed in DNA from the tumor at the primary diagnosis in 2005 (primary O^2005^) and a second-generation xenograft (xenograft^2^). A subset of potential driver mutations was identified, which provides insight into the initiating events in oligodendroglioma as well as a candidate tumor suppressor pathway on chromosome 4.

## RESULTS

### Copy number changes and copy neutral loss of heterozygosity (cnLOH) in oligodendroglioma evolution

Recurrent O^2010^ and the derived xenograft (xenograft^1^) had been previously analyzed for gross chromosomal aberrations by array comparative genomic hybridization (CGH) [13]. Array CGH revealed the characteristic genomic profiles for oligodendroglioma, which included the 1p/19q codeletion as well as chromosome 4, 6q and 14q losses that are common in higher grade oligodendrogliomas [18]. The tumor genome at a gross level appeared to be highly stable upon passage through mice, and an increase in ploidy for chromosomes 1 and 19 was not evident based on fluorescence *in situ* hybridization analysis (Miletic, H., data not shown, [19]). One major difference was a gain of the chromosomal arm 4q in xenograft^1^, which could be the result of the outgrowth of a subclone that was either present but previously undetectable in recurrent O^2010^, or it represented the generation of a new clone altogether in the mouse.

A more cryptic form of genomic instability, copy neutral loss of heterozygosity (cnLOH), has been previously described in oligodendrogliomas. In cnLOH, the chromosomes appeared to be diploid but were homozygous, indicating loss with subsequent duplication of a remaining chromosome. Because the predominant clone in recurrent O^2010^ had lost 4q, a subclone would only be distinguishable if it retained heterozygosity at 4q. Sequencing datasets for recurrent O^2010^ and xenograft^1^ were therefore analyzed for copy number and regions of heterozygosity and homozygosity. Analysis of SNVs revealed regions of homozygosity correlating with chromosomal losses on 1p, 19q, 4, 6q, and 14q in recurrent O^2010^ as expected [13]. LOH was also observed on chromosomes with two copies, such as 9p and 12. The cnLOH at 9p was previously observed in oligodendroglioma and was associated with loss of *CDKN2A* expression in approximately 30% of the cases examined [20].

**Figure 1:**
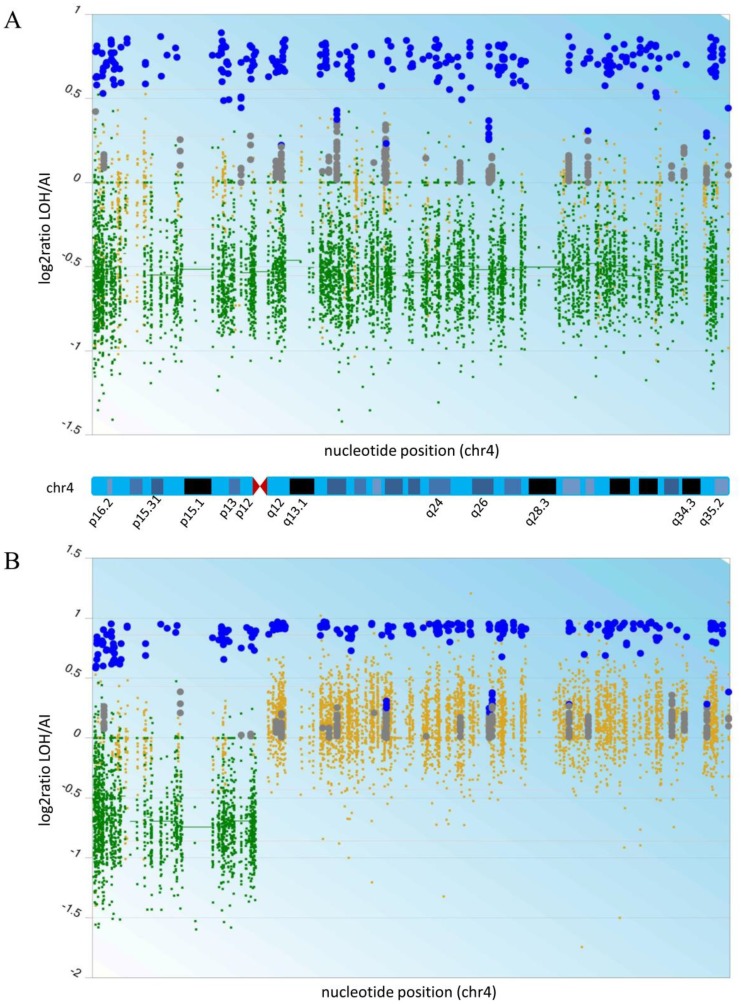
Deletion of chromosome 4 in recurrent O^2010^ (**A**) and copy neutral LOH 4q in xenograft^1^ (**B**). LOH and CNV analysis was performed using the CEQer software [21] on the whole exome sequencing data from both tumors and data from patient blood as the control. Shown is the Log2 ratio of LOH/AI data according to the formula: ratio = MaxCovAllele / (CovAllele1 + CovAllele2), whereas MaxCovAllele is the allele with the highest coverage. Small green dots indicate copy loss of CNV exons, and horizontal green bars highlight individual copy loss of CNV regions. Small yellow dots show copy neutral regions. Big blue circles indicate either a loss of heterozygosity (-1 copy loss), an allelic imbalance (copy gain) or a copy neutral imbalance. Big grey circles indicate a conserved heterozygosity.

LOH mapped similarly in the xenograft across the genome, including the cnLOH at 9p and 12. Genomic sequencing also revealed cnLOH on 4q indicating that loss of one chromosome had occurred with subsequent duplication of the remaining arm (Figure 1; [21]). Thus whether the xenograft represented a subclone from the original tumor or a new variant that developed during the engraftment process cannot be differentiated. However, these regions of cnLOH were consistent with an underlying level of genomic instability that may promote tumor development through loss of wild type and subsequent duplication of chromosomal regions harboring mutated tumor suppressor genes or oncogenes.

### Exome sequencing reveals a stable xenograft genome

We performed in-depth analysis of the exome sequencing datasets to compare the genomes of the primary tumor/xenograft pair (recurrent O^2010^/xenograft^1^) and to identify putative genes targeted by losses of chromosomes 4q, 6q, and 14q which typically occur in higher grade oligodendrogliomas [18].

Exome sequencing revealed 80 somatic variants, 73 single nucleotide variants (SNVs) and 7 small insertions and deletions (InDels) with potential effects on protein function in recurrent O^2010^. This number of variants ranks in a higher range for oligodendroglioma, but is in overall agreement with the number observed in tumors previously treated with radiation and chemotherapy, as in this case [10, 22, 23]. Of these, 71 SNVs and all 7 InDels were also present in the xenograft. The variants found in both included *IDH1* (NM_005896: c.395G>A; *R132H*), *CIC* (NM_015125: c.4421T>G; p.Val1474Gly - missense), and *FUBP1* (NM_003902:c.1307_1310delTAGA; p.Ile436fs), as previously reported. *FUBP1* on chromosome 1p sustained a small deletion, which created a truncation/frameshift [13]. Variants in tumor associated genes not typically associated with oligodendroglioma were also found, such as missense variants in *TET1* (NM_030625:c.4399G>A; p.Glu1467Lys) and *TGFBR2* (NM_003242:c.426G>C; p.Glu142Asp as well as a frameshift variant in *ARID1A* (NM_006015:c. 2290_2291insC; p.Gln766fs), a gene that is also located at 1p36 and frequently mutated in endometriosis associated ovarian cancers [24]. Variants in several genes were observed on chromosomes exhibiting homozygosity and/or loss, including *COL25A1*, *HS3ST1*, *NDST4*, and *ZCCHC4* on chromosome 4 and *ATXN2*, *NUAK1*, *RASAL1* and *ZC3H12* on chromosome 12. However, no variant or amplification was found in *PDGFRα* on 4q, a gene involved in the development of human oligodendroglioma, or in *CDKN2A* on 9p, one of the most frequently altered genes in diverse cancer types.

Only 7 novel variants were found in the xenografts. These variants could potentially be newly acquired, or they were either undetectable by our methods or in the tumor sections available. All together, these results indicate that the xenograft was genomically highly stable and reflects the genetic make-up of recurrent O^2010^.

### Deep sequencing distinguishes potential initiating events from progression events

As recurrent O^2010^ had been treated, it was of interest to identify variants present before therapy, which could help to define new driver initiating *versus* progression or passenger mutations. In addition, whether variants in the xenograft were already present either in primary O^2005^ or recurrent O^2010^ was of interest in terms of potential selective advantages during engraftment.

**Figure 2:**
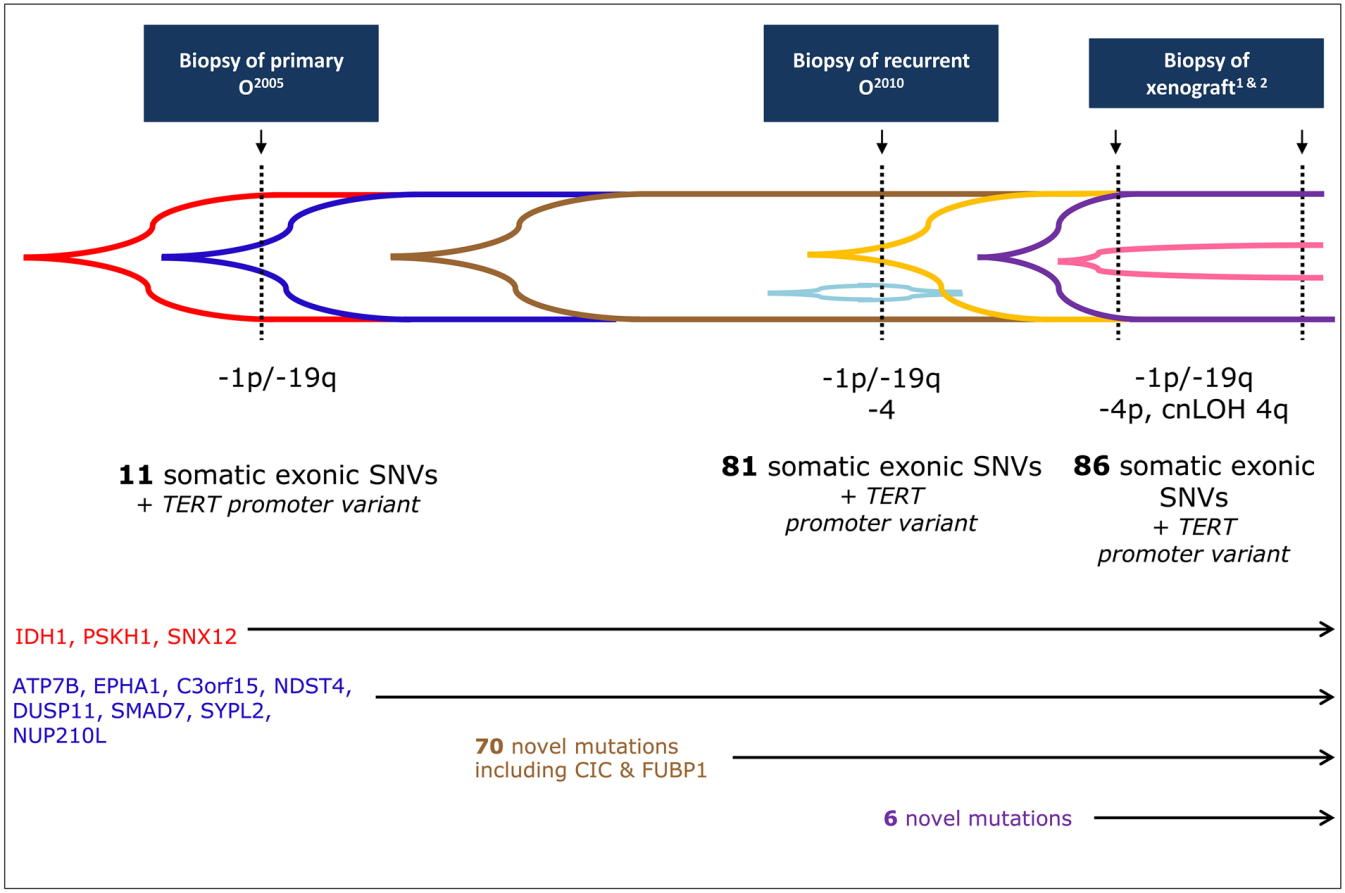
Codeletion of 1p/19q occurs before variants in *FUBP1* and *CIC*. Timeline for the acquisition of all genetic events in primary O^2005^, recurrent O^2010^, and xenografts^1&2^, including array CGH. Exome sequencing was performed on recurrent O^2010^ and xenograft^1^, and these datasets were used to generate the set of variants (total = 87) to be further analyzed by deep sequencing. Deep sequencing detected a subset of these variants in primary O^2005^ (*n* = 11) and was used to generate an allele frequency for each variant in each tumor. Only 3/11 (red) variants, including *IDH1 R132H* (NM_005896:c.395G>A), were considered to be in the majority of the cells in primary O^2005^ (allele frequency ≥ 50%). The other 8 (blue) were detectable in primary O^2005^ but at lower frequencies (< 50%). In addition to these 11 variants, 70 novel variants occurred in the recurrent O^2010^ (total = 81, 70 new, distinguished by brown, gold, and light blue). Of these, 63 were present in the majority of the cells (brown). Included are variants in *CIC* (NM_015125:c.4421T>G) and *FUBP1* (NM_003902:c.1307_1310delTAGA). A single variant, *ARHGAP6* NM_013423:c.455G>C (light blue), was found only in recurrent O^2010^. In the xenografts, only 6 new variants (purple, ≥ 50% and pink, < 50%, perhaps latest changes) appeared in addition to those from recurrent O^2010^ (*n* = 86; 6 new, 1 lost). Additional targeted Sanger sequencing revealed the presence of a variant in *TERT* promoter in all tumors.

Deep sequencing was performed for all variants with DNAs from the patient’s blood and all tumors. Deep sequencing confirmed the presence of 81/87 variants in recurrent O^2010^, while 6 variants identified originally in xenograft^1^ (out of the 7 by exome sequencing exclusively in xenograft^1^ detected ones) were not present in recurrent O^2010^ (Table 1), also not at low frequency (Figure 2).

**Table 1:** Variants exclusively found in xenografts using deep sequencing

**Position**	**Gene**	**Coding region change**	**Amino acid change**	**Allele frequency in xenograft^1^**	**Allele frequency in xenograft^2^**	**Effect**
chr10: 69748523	HERC4	NM_015601:c. 1703T>C	NM_015601:p. Val568Ala	47%	48%	missense
chr1: 179497498	AXDND1	NM_144696.4:c. 2647C>T	NM_144696.4:p. Arg883*	28%	31%	stop gained
chr13: 52440005	CCDC70	NM_031290.2:c. 491G>A	NM_031290.2:p. Trp164*	31%	34%	stop gained
chr1: 52838968	ORC1	NM_001190819.1:c. 2456C>G	NM_001190819.1:p. Ser819Cys	100%	99%	missense
chr6: 161501997	MAP3K4	NM_005922.2:c. 2182G>A	NM_005922.2:p. Glu728Lys	26%	27%	missense
chr4: 26426323	RBPJ	NM_015874:c. 706_707delAA	NM_015874:p. Lys236fs	95%	95%	frameshift

Only 11/87 somatic variants were also present in the primary O^2005^ (Table 2), so that the other mutations (*n* = 76) were considered to be due to progression and/or treatment. All variants found in primary O^2005^ were confirmed using Sanger sequencing. With the exception of one variant (one of two identified by exome sequencing), *ARHGAP6* (NM_013423:c.455G>C; p.Arg152Pro) on the X chromosome, all variants observed in recurrent O^2010^ were confirmed in both xenograft^1^ and xenograft^2^. One small InDel, *NUP210L* (NM_207308:c.2072delT; p.Leu691fs), was also detected in the DNA from the patient’s blood, but with an allele frequency of only 1.1%. This result is possibly due to a sequencing error.

**Table 2:** Variants found in primary O^2005^ using deep sequencing

Position	Gene	Coding region change	Amino acid change	Allele frequency	Effect
chr1: 110019535	SYPL2	NM_001040709:c. 392C>G	NM_001040709:p.Ala131Gly	19.8%	missense
chr13: 52539048	ATP7B	NM_000053:c.1829C>T	NM_000053:p.Pro610Leu	3%	missense
chr16: 67943302	PSKH1	NM_006742:c.650G>A	NM_006742:p.Arg217Gln	63%	missense
chr2: 209113112	IDH1	NM_005896:c.395G>A	NM_005896:p.Arg132His	37%	missense
chr2: 73996452	DUSP11	NM_003584:c.575A>G	NM_003584:p.Asp192Gly	20%	missense
chr3: 119462961	C3orf15	NM_033364:c.1820G>A	NM_033364:p.Arg607Gln	11%	missense
chr4: 115754766	NDST4	NM_022569:c.2392C>G	NM_022569:p.Leu798Val	17.5%	missense
chr7: 143095768	EPHA1	NM_005232:c.1262C>A	NM_005232:p.Ala421Asp	10%	missense
chrX: 70280873	SNX12	NM_001256188:c.470G>A	NM_001256188:p.Arg157His	56%	missense
chr1: 154067525	NUP210L	NM_207308:c.2072delT	NM_207308:p.Leu691fs	1.2%	frameshift
chr18: 46447974	SMAD7	NM_001190823:c.484_485insT	NM_001190823:p.Pro162fs	8.3	frameshift

Of the genes already associated with the development of oligodendroglioma (*IDH1/IDH2*, *CIC*, and *FUBP1*), remarkably only the mutation in *IDH1* (NM_005896:c.395G>A) was present in the primary O^2005^. *FUBP1* and *CIC* variants were not present, even though 1p/19q codeletion had been detected by array CGH in primary O^2005^ (Supplementary Figure 1). Thus, *FUBP1* and *CIC* variants appeared to occur secondary to the losses of 1p and 19q during tumor progression and were not necessary changes for tumor initiation in this case.

In addition to the *FUBP1* frameshift variant, we also found a loss-of-function mutation of the known tumor suppressor gene *ARID1A* on 1p in the recurrent O^2010^ but not in primary O^2005^. In the TCGA dataset, alterations were detected in *ARID1A* in low grade glioma (SNV: 3.9%, 11/283; deletion: 0.4%, 1/283) and glioblastoma (SNV: 0.7%, 2/281) [25, 26]. As this variant emerged following treatment of the patient, the relationship to tumor development remains unclear. *CIC*, *FUBP1*, and *ARID1A* variants were all maintained through xenograft^2^ and in a homozygous state.

### The TERT promoter is mutated

Most variants in the *TERT* promotor occurring in human gliomas are one of two changes: NM_198253.2:c.-124G>A (C228T) and NM_198253.2: c.-146G>A (C250T) located in a common SNV *rs2853669* [12, 27, 28]. In oligodendrogliomas, a variant in the *TERT* promoter was found in 78% of cases (*n* = 45), and of these, 43% were the C250T variant [12]. To determine whether the *TERT* promoter also harbored a variant in our system, we performed targeted Sanger sequencing on DNA from each of the tumor samples. C250T (NM_198253.2: c.-146G>A; p.Cys250Thr) was found in all samples and was heterozygous.

### Temporal acquisition of variants reconstructed based on allele frequency

**Figure 3:**
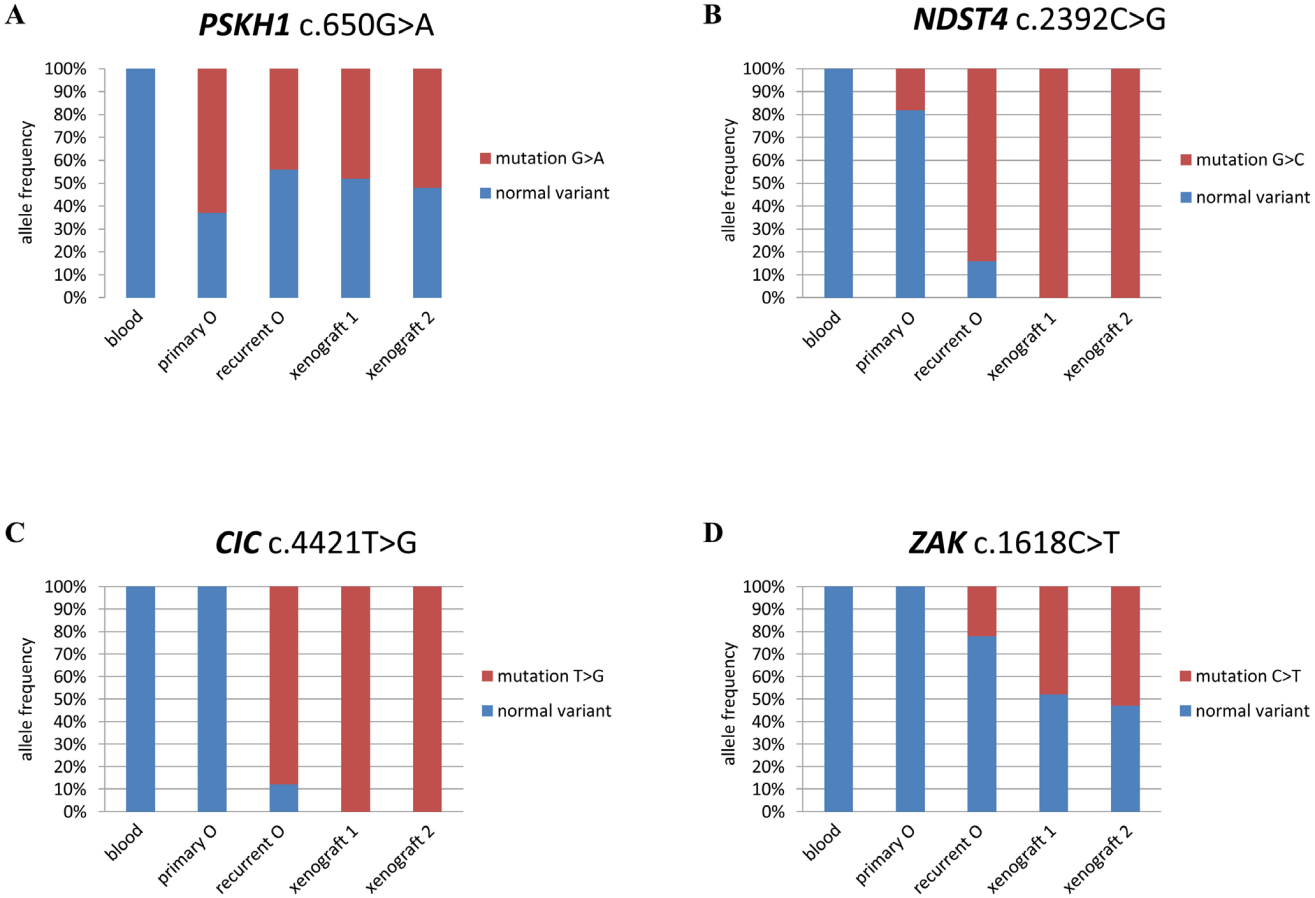
Allele frequency distinguishes between early and late variants. Graphic representation of allele frequency (% of reads for variant indicated) for variants in 4 genes in the tissue sample indicated. (**A**) *PSKH1*; (**B**) *NDST4*; (**C**) *CIC*; and (**D**) *ZAK*.

Allele frequency based on the sequencing reads was also used to more precisely reconstruct the temporal acquisition of variants (Figures 2 and 3). We identified 10 variants that displayed a significantly lower frequency where the variant first appeared, than in the tumor that followed. Eight were variants that were already detectable in primary O^2005^: the missense variants in *ATP7B* (NM_000053:c.1829C>T; allele frequency, AF 3%); *C3orf15* (NM_033364:c.1820G>A; AF, 10%); *DUSP11* (NM_003584:c.575A>G; AF, 10%); *EPHA1* (NM_005232:c.1262C>A; AF 10%); *NDST4* (NM_022569:c.2392C>G; AF 20%); *SYPL2* (NM_001040709:c.392C>G; AF 20%) and the frame shift mutations in *NUP210L* (NM_207308:c. 2072delT; AF 1%) and *SMAD7* (NM_001190823:c.484_485insT; AF 10%). The remaining two variants initially appeared in recurrent O^2010^ and became predominant in xenograft^1^: *ZAK* (NM_016653:c.1618C>T; p.Gln540*; AF 20%; Figure 3) and the InDel in *KPNA1* (NM_002264:c.179_181delAAG; p.lu60del; AF 20%). Only two variants, *PSKH1* (NM_006742.2:c.650G>A; p.Arg217Gln), and *SNX12* (NM_001256188:c.470G>A; p.Arg157His) were present in ≥ 50% of reads as early as primary O^2005^. Variants in these genes and *IDH1* exhibited an allele frequency that remained stable across all tumor samples. They were considered to be heterozygous and uniformly distributed amongst tumor cells, indicating that they had occurred early in tumor development.

Variants that exhibited an allele frequency of < 50% were considered to have occurred later and in a subset of tumor cells. Any increase in allele frequency could be an indication that a subclone bearing the variant had been selected for. Examples of such variants were *NDST4* (NM_022569:c.2392C>G; p.Leu798Val) and *ZAK* (NM_016653:c.1618C>T; p.Gln540*), which were first detected in primary O^2005^ and recurrent O^2010^, respectively (Figure 3). *NDST4* represented an interesting case because of the changing status of chromosome arm 4q. In primary O^2005^, where 4q was diploid, the mutation was detected at a frequency of ~20%. In recurrent O^2010^, the allele frequency of the *NDST4* variant was 80%. This result was consistent with selection of tumor cells harboring reduction to homozygosity at *NDST4*/chromosome arm 4q. The allele frequency of the variant increased to 100% in xenograft^1^ in the absence of contaminating non-neoplastic human cells. In xenograft^1^ with cnLOH on the 4q arm, a normal variant of *NDST4* was no longer present, indicating that duplication of the remaining mutated allele had occurred.

### NDST4 on 4q is not frequently mutated in human oligodendrogliomas

Chromosomal losses 4q and 14q have been associated with higher grade oligodendroglioma. *NDST4* was a candidate tumor suppressor gene on chromosome 4 as the *NDST4* previous variant was already detectable in the primary tumor and the wild-type allele was lost in recurrent O^2010^, indicating a loss of function mechanism. To investigate if alterations in *NDST4* might play a role in the development of oligodendroglioma, we performed Sanger sequencing to analyze all coding exons of the gene in our own cohort of primary oligodendrogliomas (*n* = 15). We found no variants in coding regions of *NDST4* in this tumor set; only known polymorphisms and variants in intronic sequences were observed. However, only 3 cases in our cohort had sustained loss of chromosome 4. In TCGA datasets, 8 missense mutations and deletions in *NDST4* were found in lower grade gliomas, while 17 missense and 1 truncating variant were found in glioblastoma [25, 26]. All together the somatic mutation frequency was 0.6% [25, 26]. Thus, although *NDST4* remains a gene of interest in tumor development and indeed might play a role in the progression from primary O^2005^ to recurrent O^2010^, *NDST4* mutation seems to be a rare event in oligodendroglioma.

## DISCUSSION

Exome sequencing coupled with array CGH performed on a rare oligodendroglioma xenograft revealed a highly stable genome relative to the recurrent human tumor from which it was derived. Using deep sequencing (and targeted Sanger sequencing for the *TERT* promoter), we identified a set of early and potential driver variants (*n* = 12) already present in genes in primary O^2005^ which included *IDH1* and *TERT*, but surprisingly not *FUBP1* and *CIC*. Allele frequencies for 10 of these 12 variants was ≤ 50%, but increased for some variants indicating possible selection of tumor cell subpopulations in response to normal progression, treatment, or engraftment. Finally, the increase in allele frequency of the *NDST4* variant in the tumor series was consistent with the selection of a tumor suppressor gene.

The overall number of variants (*n* = 82 - including *TERT*) in recurrent O^2010^ was significantly higher compared to primary O^2005^ (*n* = 12 - including *TERT*) and the average for primary oligodendrogliomas, but was consistent with cases from patients who had undergone treatment [10, 11]. All variants were also detected in xenograft^1^, along with 6 new changes including 5 SNVs and 1 small InDel. These 6 changes in xenograft^1^ might have spontaneously occurred during progression in xenograft^1^ or were previously undetectable but selected for during engraftment into the mouse. Only one variant in *ARHGAP6* was exclusively found in recurrent O^2010^ but at a low allele frequency (12%).

Through the deep sequencing analysis emerged a sequence of mutational events that may be more generally relevant to the development of a subset of human oligodendrogliomas. *FUBP1* and *CIC* variants first appeared in recurrent O^2010^. Thus, the scenario for inactivation of these tumor suppressor genes in some cases may be loss of heterozygosity before selection of cells with variants. It has been previously suggested that haploinsufficiency at 1p is tumor promoting [29]. In oligodendrogliomas, haploinsufficiency occurs with a high frequency in the context of an *IDH1/IDH2* variant [3]. In such cases, loss in combination with epigenetic modification may sufficiently knock down expression of key target genes to promote uncontrolled proliferation and/or inhibit differentiation. Variants in *FUBP1* and *CIC* may further stabilize the phenotype established by haploinsufficiency along with epigenetic changes.

In support of this scenario, results from several studies indicate that concurrent variants in *FUBP1* and *CIC* appear infrequently. In two early studies, only ~10% of human oligodendrogliomas with codeletion of 1p and 19q were found to harbor simultaneous variants [10, 22]. Our own study from 2013 corroborated these results. *FUBP1* and *CIC* were both mutated in only 12% (2/17) of oligodendrogliomas examined, and all had sustained a variant in *IDH1* [30]. In the dataset from the TCGA, simultaneous variants occurred in a higher proportion of tumors, 24% (13/53) [25, 26]. However, while we used deep sequencing in our analysis, there are other possibilities that might account for the low frequency of simultaneous mutations in primary tumors, such as technique and sample bias due to tumor heterogeneity or small undetectable subclones.

Although ultimately it is the accumulation of genetic events that results in cancer, the putative timeline of our oligodendroglioma might provide insight for the contribution of *IDH*, *TERT* and 1p/19 codeletion to the development of oligodendroglioma. First, the results might support studies indicating that *IDH* mutation occurs early in a stem/progenitor cell type with subsequent *p53* mutations or 1p/19 codeletion defining the path to astrocytoma or oligodendroglioma. *IDH* mutation appears to be a gatekeeper, triggering uncontrolled proliferation which renders cells vulnerable to additional tumor promoting genetic events [31]. Second, 1p/19q codeletion might drive the development of oligodendroglioma through mechanisms other than loss of *FUBP1* and *CIC* [32]. Examination of expression datasets for oligodendroglioma have led to the identification of a network of genes in the codeleted region of 1p/19q that may promote the development of oligodendroglioma. Finally, we can wonder whether *TERT* or *IDH* mutations have a more direct impact on genomic instability. *TERT* promoter mutations have been shown to extend cellular lifespan but do not necessarily prevent shortening of telomeres [33]. These shorter telomeres however later in tumor development contribute to genomic instability. A second possibility is mutation of the *IDH* gene itself. *IDH* mutation promotes epigenetic modification of DNA, and methylation has been implicated in chromosomal instability, although more generally through global hypomethylation [34]. The fact that the incidence of *IDH* mutation is so high in 1p/19q codeleted oligodendrogliomas and that cnLOH is also commonly observed further supports this possible role for *IDH* mutation in cancer [3, 20].

Finally, deep sequencing performed on primary O^2005^ importantly also enabled us to distinguish potential driver from passenger variants in recurrent O^2010^. *NDST4* on chromosome 4q emerged as a potential tumor suppressor gene as it was one of the few genes with a variant already detectable in primary O^2005^. Unlike *IDH1* or *PSKH1*, this variant was probably present only in a subpopulation of cells in primary O^2005^ which later became predominant. The protein is involved in heparan sulfate synthesis, and the distribution of heparan sulfate is altered in tumorigenesis. Heparan sulfate synthesis has also been shown to be necessary for growth factor activity. Furthermore, heparan sulfates contribute to cell-cell and cell-extracellular matrix interactions forming barriers for tumor invasion [35]. Interestingly, two additional genes mutated in recurrent O^2010^, *EXTL3* (chromosome 8) and *HS3ST1* (chromosome 4), are also involved in heparan sulfate synthesis. These genes are altered in many cancers and like *NDST4*, frequent alterations are present in skin cutaneous melanoma and lung cancer [25, 26]. Therefore, alteration of heparan sulfate synthesis could be important in tumor biology.

In conclusion, we have used exome sequencing datasets from a unique series of a recurrent human tumor and the derived xenografts to construct an evolutionary genetic profile. Although the primary oligodendroglioma exhibited 1p/19q codeletion, the variants detected did not include changes in *FUBP1* and *CIC*. Only a few variants, in addition to *IDH1* R132H and *TERT* C250T, were observed in the primary tumor that gained predominance and might represent interesting candidate genes for oligodendroglioma development. Because mutations in several of these genes are rare in oligodendrogliomas, and human cancer in general, some of these variants may simply represent hits in the so-called “hills” of the oligodendroglioma genome landscape [31]. Functional experiments are therefore required to determine whether the genes identified in this study are in fact true driver genes in human cancer.

## MATERIALS AND METHODS

### Ethics statement

Patient material was obtained from surgeries performed at the Haukeland University Hospital (Bergen, Norway). Written consent was obtained from all patients to use tissues and clinical data for research purposes. Procedures were approved for the project (project number 013.09) by the Regional Ethics Committee (Bergen, Norway). All animal protocols were approved by authorities in an AAALAC accredited facility at the Haukeland University Hospital and in accordance with the national regulations of Norway (project numbers 2010 2658 and 2011 3079).

### Patient and xenograft tumors

Tissue was obtained from a recurrent primary tumor from 2010 (oligodendroglioma WHO grade III; recurrent O^2010^), and implanted as previously described [13]. Xenograft tissue was harvested at the first implantation to obtain the first-generation xenograft (xenograft^1^). Cells from this tumor were reimplanted to obtain the second-generation xenograft (xenograft^2^). Portions of all three tumors were harvested and stored at –80°C for molecular analysis. Tissue from the original tumor (oligodendroglioma WHO grade III; primary O^2005^) was obtained from archived paraffin embedded surgical material in 2005.

### DNA isolation

DNAs were isolated from patient material that had been immediately frozen in liquid nitrogen subsequent to surgery. Frozen sections were prepared, and every fifteenth section was stained with H&E to ensure > 60% tumor cell content. As a control, DNA was obtained from frozen patient blood (–20°C) by isolating nuclei with Buffer C1 from the Genomic DNA Buffers Kit (Qiagen; Germantown, MD, USA). Samples were treated with proteinase K overnight at 50°C in ATL buffer (Qiagen) and then DNAse free RNAseA (Fermentas/Thermo Fisher Scientific; Waltham, MA, USA) for 5 min at room temperature. Reactions were extracted with phenol:chloroform:isoamyl alcohol 25:24:1 saturated with 10 mM tris, pH 8.0, 1 mM EDTA (Sigma; St. Louis, MO, USA) and precipitated in 2.5M NH_4_OAc and 2.5 volumes of 100% ethanol. DNAs were resuspended in Nuclease-Free water (Qiagen). DNA was extracted from FFPE tissue samples using the QIAamp DNA FFPE Tissue Kit (Qiagen) according to the manufacturer’s protocol.

### Exome sequencing

Agilent SureSelect target enrichment kit (SureSelect Human All Exon 50 Mb kit, Agilent; Santa Clara, CA, USA) was used for exome library construction according to the manufacturer’s protocol. Captured DNA libraries were sequenced on the Illumina HiSeq2000 platform at a coverage of 100× (Illumina; San Diego, CA, USA).

### Data analysis

Briefly, the genomes derived from human tissues were aligned against the human genome hg19. Analysis of the sequencing reads from the xenografts underwent a cleaning process to eliminate contaminating mouse reads. Two alignment steps were performed. In the first step, the sequencing reads were aligned against the human (hg19) and the mouse (mm10) genomes separately. Reads with better mapping results (higher mapping quality) in the mouse genome than in the human genome were deleted. In the second step, surviving reads were aligned against a chimeric genome, which was obtained through the concatenation of the human and mouse genomes. The resulting BAM file was then separated into those reads that mapped to the human or the mouse genome using SAMtools (version 0.1.18) [36, 37]. The cleaning process was therefore accomplished by cross-referencing reads with a high mapping quality in the other set and eliminating them.

The pipeline for all the samples was the following: alignment using BWA (version 0.7.2) [38], and post-alignment processing using GATK [39] for local realignment, duplicate removal and score recalibration, and finally variant calling between each pair of samples using VarScan (version 2.3.2) [40]. SAMtools (version 0.1.18) was used to create the needed pileup file [36, 37]. False positives were eliminated by a false positive filtering (strandedness (0.1); maximum mismatch quality sum (100); minimum average distance to effective 3’end (0.1) [40].

After a statistical filtering for somatic variants (for details see Supplementary Material), we selected for mutation type (non-synonymous substitution, start and stop codon gain or loss, and frame-shifts), to focus on variants with potential effects at the protein level. All called variants were hand-curated, which included an evaluation using the Integrative Genomics Viewer provided by the Broad Institute [41, 42], applying the BLAT - BLAST- like Alignment Tool [43] of the UCSC Genome Browser [44], and including additional information from the UCSC Genome Browser, such as pseudogenes and self-chain or repetitive regions. Variants were determined to be damaging/deleterious and disease causing by several prediction software programs, including PolyPhen-2, Mutation Taster, Mutation Assessor, PROVEAN and SIFT [45–51]. Details of the variant selection process are described in Supplementary Materials.

### PCR and targeted sequencing

All variants were additionally verified using Sanger and/or deep sequencing. Primers were designed against regions of interest using Primer3 software (version 4.1.0) [52, 53]. Primers and conditions used are shown in Supplementary Data. A first/preliminary analysis of the xenograft sequencing data revealed a large number of novel variants that were suspected to be due to contamination with mouse tissue rather than novel variants occurring during engraftment. Therefore, for validation of variants, we designed all primers to bind to human sequences only. Validation of primers was performed on normal human control DNA obtained from blood and DNA from mouse tissue to verify that primers would only yield a PCR-product in human but not mouse DNA.

For ultra-deep sequencing, PCR was carried out using a proofreading polymerase (Phusion^®^ Hot Start Flex DNA Polymerase; New England Biolabs; Ipswich, MA, USA) according to the manufacturer’s instructions under the following cycling conditions: initial denaturation step: 98°C, 30 sec; amplification step (34×): 98°C, 10 sec, 61.5 – 66.5°C, 30 sec, 72°C, 30 sec; final extension: 72°C, 10 min. The correct size of PCR products was confirmed by agarose gel electrophoresis (2.0% agarose impregnated with GelRed; 1 h, 110 V) and purified from contaminating nucleotides and primers by PEG precipitation. Libraries were sequenced to an average coverage of 36,000× on an Illumina HiSeq2000 Next Generation Sequencer (Illumina; San Diego, CA, USA). Results were analyzed using CLC Genomics Workbench Version 7.5. Reads were aligned to the human reference genome (hg19) and variants were called using the Basic Variant Detection algorithm (minimum coverage 1000×; minimum count 2, minimum frequency 1%; the base quality filter was used with default values, no direction or position filters were applied).

Variants found in primary O^2005^ were validated using Sanger’s dye terminator method on an ABI 3130x1 DNA Analyzer (Applied Biosystems/Thermo Fisher Scientific). Products were cleaned with rAPid alkaline phosphatase (Roche Diagnostics GmbH; Mannheim, Germany) and exonuclease I treatment (New England Biolabs) at 37°C for 1 h followed by inactivation at 80°C for 20 min.

### Array comparative genomic hybridization

Array CGH was performed on Agilent’s SurePrint G3 Human CGH Microarray Kit 2x400K (Design ID021850, Agilent, Santa Clara, CA, USA) according to the manufacturer’s protocol. Scanning of the hybridized arrays was carried out using the Agilent High-Resolution Microarray scanner. Raw data were processed by the Feature Extraction 9.5 (Agilent) software and normalization was performed using the default settings. Feature Extraction files were imported into Genomic Workbench 7.0 (Agilent Technologies) for visualization and analysis. After diploid centralization and GC correction, aberrations were called using the ADM2 algorithm with a threshold setting of 20, centralization on with threshold of 25 and an aberration filter min Probes = 5 and minAvgAbsLogRatio = 0.35 for amplifications and deletions [13].

### The Cancer Genome Atlas (TCGA) dataset analyses

TCGA data was analyzed using the cbioPortal for Cancer Genomics [25, 26].

## SUPPLEMENTARY MATERIALS



## Abbreviations

AF: allele frequency
CGH: comparative genomic hybridization
cnLOH: copy neutral loss of heterozygosity
InDel: insertions/deletions
LOH: loss of heterozygosity
O: oligodendroglioma
SNV: single nucleotide variants.

## References

[1] Parsons DW, Jones S, Zhang X, Lin JC, Leary RJ, Angenendt P, Mankoo P, Carter H, Siu IM, Gallia GL, Olivi A, McLendon R, Rasheed BA, et al. An integrated genomic analysis of human glioblastoma multiforme. Science. 2008; 321:1807–12. 10.1126/science.1164382. 18772396 PMC2820389

[2] Balss J, Meyer J, Mueller W, Korshunov A, Hartmann C, von Deimling A. Analysis of the IDH1 codon 132 mutation in brain tumors. Acta Neuropathol. 2008; 116:597–602. 10.1007/s00401-008-0455-2. 18985363

[3] Yan H, Parsons DW, Jin G, McLendon R, Rasheed BA, Yuan W, Kos I, Batinic-Haberle I, Jones S, Riggins GJ, Friedman H, Friedman A, Reardon D, et al. IDH1 and IDH2 mutations in gliomas. N Engl J Med. 2009; 360:765–73. 10.1056/NEJMoa0808710. 19228619 PMC2820383

[4] Dang L, White DW, Gross S, Bennett BD, Bittinger MA, Driggers EM, Fantin VR, Jang HG, Jin S, Keenan MC, Marks KM, Prins RM, Ward PS, et al. Cancer-associated IDH1 mutations produce 2-hydroxyglutarate. Nature. 2009; 462:739–44. 10.1038/nature08617. 19935646 PMC2818760

[5] Figueroa ME, Abdel-Wahab O, Lu C, Ward PS, Patel J, Shih A, Li Y, Bhagwat N, Vasanthakumar A, Fernandez HF, Tallman MS, Sun Z, Wolniak K, et al. Leukemic IDH1 and IDH2 mutations result in a hypermethylation phenotype, disrupt TET2 function, and impair hematopoietic differentiation. Cancer Cell. 2010; 18:553–67. 10.1016/j.ccr.2010.11.015. 21130701 PMC4105845

[6] Lu C, Ward PS, Kapoor GS, Rohle D, Turcan S, Abdel-Wahab O, Edwards CR, Khanin R, Figueroa ME, Melnick A, Wellen KE, O’Rourke DM, Berger SL, et al. IDH mutation impairs histone demethylation and results in a block to cell differentiation. Nature. 2012; 483:474–8. 10.1038/nature10860. 22343901 PMC3478770

[7] Amary MF, Bacsi K, Maggiani F, Damato S, Halai D, Berisha F, Pollock R, O’Donnell P, Grigoriadis A, Diss T, Eskandarpour M, Presneau N, Hogendoorn PC, et al. IDH1 and IDH2 mutations are frequent events in central chondrosarcoma and central and periosteal chondromas but not in other mesenchymal tumours. J Pathol. 2011; 224:334–43. 10.1002/path.2913. 21598255

[8] Amary MF, Damato S, Halai D, Eskandarpour M, Berisha F, Bonar F, McCarthy S, Fantin VR, Straley KS, Lobo S, Aston W, Green CL, Gale RE, et al. Ollier disease and Maffucci syndrome are caused by somatic mosaic mutations of IDH1 and IDH2. Nat Genet. 2011; 43:1262–5. 10.1038/ng.994. 22057236

[9] Mardis ER, Ding L, Dooling DJ, Larson DE, McLellan MD, Chen K, Koboldt DC, Fulton RS, Delehaunty KD, McGrath SD, Fulton LA, Locke DP, Magrini VJ, et al. Recurring mutations found by sequencing an acute myeloid leukemia genome. N Engl J Med. 2009; 361:1058–66. 10.1056/NEJMoa0903840. 19657110 PMC3201812

[10] Bettegowda C, Agrawal N, Jiao Y, Sausen M, Wood LD, Hruban RH, Rodriguez FJ, Cahill DP, McLendon R, Riggins G, Velculescu VE, Oba-Shinjo SM, Marie SK, et al. Mutations in CIC and FUBP1 contribute to human oligodendroglioma. Science. 2011; 333:1453–5. 10.1126/science.1210557. 21817013 PMC3170506

[11] Yip S, Butterfield YS, Morozova O, Chittaranjan S, Blough MD, An J, Birol I, Chesnelong C, Chiu R, Chuah E, Corbett R, Docking R, Firme M, et al. Concurrent CIC mutations, IDH mutations, and 1p/19q loss distinguish oligodendrogliomas from other cancers. J Pathol. 2012; 226:7–16. 10.1002/path.2995. 22072542 PMC3246739

[12] Killela PJ, Reitman ZJ, Jiao Y, Bettegowda C, Agrawal N, Diaz LA Jr, Friedman AH, Friedman H, Gallia GL, Giovanella BC, Grollman AP, He TC, He Y, et al. TERT promoter mutations occur frequently in gliomas and a subset of tumors derived from cells with low rates of self-renewal. Proc Natl Acad Sci U S A. 2013; 110:6021–6. 10.1073/pnas.1303607110. 23530248 PMC3625331

[13] Klink B, Miletic H, Stieber D, Huszthy PC, Campos Valenzuela JA, Balss J, Wang J, Schubert M, Sakariassen PO, Sundstrom T, Torsvik A, Aarhus M, Mahesparan R, et al. A novel, diffusely infiltrative xenograft model of human anaplastic oligodendroglioma with mutations in FUBP1, CIC, and IDH1. PLoS One. 2013; 8:e59773. 10.1371/journal.pone.0059773. 23527265 PMC3602110

[14] Kelly JJ, Blough MD, Stechishin OD, Chan JA, Beauchamp D, Perizzolo M, Demetrick DJ, Steele L, Auer RN, Hader WJ, Westgate M, Parney IF, Jenkins R, et al. Oligodendroglioma cell lines containing t(1;19)(q10;p10). Neuro Oncol. 2010; 12:745–55. 10.1093/neuonc/noq031. 20388696 PMC2940664

[15] Wakimoto H, Tanaka S, Curry WT, Loebel F, Zhao D, Tateishi K, Chen J, Klofas LK, Lelic N, Kim JC, Dias-Santagata D, Ellisen LW, Borger DR, et al. Targetable signaling pathway mutations are associated with malignant phenotype in IDH-mutant gliomas. Clin Cancer Res. 2014; 20:2898–909. 10.1158/1078-0432.CCR-13-3052. 24714777 PMC4070445

[16] Srikanth M, Kim J, Das S, Kessler JA. BMP signaling induces astrocytic differentiation of clinically derived oligodendroglioma propagating cells. Mol Cancer Res. 2014; 12:283–94. 10.1158/1541-7786.MCR-13-0349. 24269952 PMC4006982

[17] Navis AC, Niclou SP, Fack F, Stieber D, van Lith S, Verrijp K, Wright A, Stauber J, Tops B, Otte-Holler I, Wevers RA, van Rooij A, Pusch S, et al. Increased mitochondrial activity in a novel IDH1-R132H mutant human oligodendroglioma xenograft model: *in situ* detection of 2-HG and α-KG. Acta Neuropathol Commun. 2013; 1:18. 10.1186/2051-5960-1-18. 24252742 PMC3893588

[18] Kitange G, Misra A, Law M, Passe S, Kollmeyer TM, Maurer M, Ballman K, Feuerstein BG, Jenkins RB. Chromosomal imbalances detected by array comparative genomic hybridization in human oligodendrogliomas and mixed oligoastrocytomas. Genes Chromosomes Cancer. 2005; 42:68–77. 10.1002/gcc.20108. 15472895

[19] Nigro JM, Takahashi MA, Ginzinger DG, Law M, Passe S, Jenkins RB, Aldape K. Detection of 1p and 19q loss in oligodendroglioma by quantitative microsatellite analysis, a real-time quantitative polymerase chain reaction assay. Am J Pathol. 2001; 158:1253–62. 10.1016/S0002-9440(10)64076-X. 11290543 PMC1891922

[20] Idbaih A, Ducray F, Dehais C, Courdy C, Carpentier C, de Bernard S, Uro-Coste E, Mokhtari K, Jouvet A, Honnorat J, Chinot O, Ramirez C, Beauchesne P, et al, and POLA Network. SNP array analysis reveals novel genomic abnormalities including copy neutral loss of heterozygosity in anaplastic oligodendrogliomas. PLoS One. 2012; 7:e45950. 10.1371/journal.pone.0045950. 23071531 PMC3468603

[21] Piazza R, Magistroni V, Pirola A, Redaelli S, Spinelli R, Redaelli S, Galbiati M, Valletta S, Giudici G, Cazzaniga G, Gambacorti-Passerini C. CEQer: a graphical tool for copy number and allelic imbalance detection from whole-exome sequencing data. PLoS One. 2013; 8:e74825. 10.1371/journal.pone.0074825. 24124457 PMC3790773

[22] Jiao Y, Killela PJ, Reitman ZJ, Rasheed AB, Heaphy CM, de Wilde RF, Rodriguez FJ, Rosemberg S, Oba-Shinjo SM, Nagahashi Marie SK, Bettegowda C, Agrawal N, Lipp E, et al. Frequent ATRX, CIC, FUBP1 and IDH1 mutations refine the classification of malignant gliomas. Oncotarget. 2012; 3:709–22. 10.18632/oncotarget.588. 22869205 PMC3443254

[23] Johnson BE, Mazor T, Hong C, Barnes M, Aihara K, McLean CY, Fouse SD, Yamamoto S, Ueda H, Tatsuno K, Asthana S, Jalbert LE, Nelson SJ, et al. Mutational analysis reveals the origin and therapy-driven evolution of recurrent glioma. Science. 2014; 343:189–93. 10.1126/science.1239947. 24336570 PMC3998672

[24] Wiegand KC, Shah SP, Al-Agha OM, Zhao Y, Tse K, Zeng T, Senz J, McConechy MK, Anglesio MS, Kalloger SE, Yang W, Heravi-Moussavi A, Giuliany R, et al. ARID1A mutations in endometriosis-associated ovarian carcinomas. N Engl J Med. 2010; 363:1532–43. 10.1056/NEJMoa1008433. 20942669 PMC2976679

[25] Gao J, Aksoy BA, Dogrusoz U, Dresdner G, Gross B, Sumer SO, Sun Y, Jacobsen A, Sinha R, Larsson E, Cerami E, Sander C, Schultz N. Integrative analysis of complex cancer genomics and clinical profiles using the cBioPortal. Sci Signal. 2013; 6:pl1. 10.1126/scisignal.2004088. 23550210 PMC4160307

[26] Cerami E, Gao J, Dogrusoz U, Gross BE, Sumer SO, Aksoy BA, Jacobsen A, Byrne CJ, Heuer ML, Larsson E, Antipin Y, Reva B, Goldberg AP, et al. The cBio cancer genomics portal: an open platform for exploring multidimensional cancer genomics data. Cancer Discov. 2012; 2:401–4. 10.1158/2159-8290.CD-12-0095. 22588877 PMC3956037

[27] Borah S, Xi L, Zaug AJ, Powell NM, Dancik GM, Cohen SB, Costello JC, Theodorescu D, Cech TR. Cancer. TERT promoter mutations and telomerase reactivation in urothelial cancer. Science. 2015; 347:1006–10. 10.1126/science.1260200. 25722414 PMC4640672

[28] Vinagre J, Almeida A, Populo H, Batista R, Lyra J, Pinto V, Coelho R, Celestino R, Prazeres H, Lima L, Melo M, da Rocha AG, Preto A, et al. Frequency of TERT promoter mutations in human cancers. Nat Commun. 2013; 4:2185. 10.1038/ncomms3185. 23887589

[29] Henrich KO, Schwab M, Westermann F. 1p36 tumor suppression--a matter of dosage? Cancer Res. 2012; 72:6079–88. 10.1158/0008-5472.CAN-12-2230. 23172308

[30] Eisenreich S, Abou-El-Ardat K, Szafranski K, Campos Valenzuela JA, Rump A, Nigro JM, Bjerkvig R, Gerlach EM, Hackmann K, Schrock E, Krex D, Kaderali L, Schackert G, et al. Novel CIC point mutations and an exon-spanning, homozygous deletion identified in oligodendroglial tumors by a comprehensive genomic approach including transcriptome sequencing. PLoS One. 2013; 8:e76623. 10.1371/journal.pone.0076623. 24086756 PMC3785522

[31] Vogelstein B, Papadopoulos N, Velculescu VE, Zhou S, Diaz LA Jr, Kinzler KW. Cancer genome landscapes. Science. 2013; 339:1546–58. 10.1126/science.1235122. 23539594 PMC3749880

[32] Gladitz J, Klink B, Seifert M. Network-based analysis of oligodendrogliomas predicts novel cancer gene candidates within the region of the 1p/19q co-deletion. Acta Neuropathol Commun. 2018; 6:49. 10.1186/s40478-018-0544-y. 29890994 PMC5996550

[33] Chiba K, Lorbeer FK, Shain AH, McSwiggen DT, Schruf E, Oh A, Ryu J, Darzacq X, Bastian BC, Hockemeyer D. Mutations in the promoter of the telomerase gene TERT contribute to tumorigenesis by a two-step mechanism. Science. 2017; 357:1416–20. 10.1126/science.aao0535. 28818973 PMC5942222

[34] Eden A, Gaudet F, Waghmare A, Jaenisch R. Chromosomal instability and tumors promoted by DNA hypomethylation. Science. 2003; 300:455. 10.1126/science.1083557. 12702868

[35] Tzeng ST, Tsai MH, Chen CL, Lee JX, Jao TM, Yu SL, Yen SJ, Yang YC. NDST4 is a novel candidate tumor suppressor gene at chromosome 4q26 and its genetic loss predicts adverse prognosis in colorectal cancer. PLoS One. 2013; 8:e67040. 10.1371/journal.pone.0067040. 23825612 PMC3692540

[36] Li H. A statistical framework for SNP calling, mutation discovery, association mapping and population genetical parameter estimation from sequencing data. Bioinformatics. 2011; 27:2987–93. 10.1093/bioinformatics/btr509. 21903627 PMC3198575

[37] Li H, Durbin R. Fast and accurate long-read alignment with Burrows-Wheeler transform. Bioinformatics. 2010; 26:589–95. 10.1093/bioinformatics/btp698. 20080505 PMC2828108

[38] Li H, Handsaker B, Wysoker A, Fennell T, Ruan J, Homer N, Marth G, Abecasis G, Durbin R, and 1000 Genome Project Data Processing Subgroup. The Sequence Alignment/Map format and SAMtools. Bioinformatics. 2009; 25:2078–9. 10.1093/bioinformatics/btp352. 19505943 PMC2723002

[39] McKenna A, Hanna M, Banks E, Sivachenko A, Cibulskis K, Kernytsky A, Garimella K, Altshuler D, Gabriel S, Daly M, DePristo MA. The Genome Analysis Toolkit: a MapReduce framework for analyzing next-generation DNA sequencing data. Genome Res. 2010; 20:1297–303. 10.1101/gr.107524.110. 20644199 PMC2928508

[40] Koboldt DC, Zhang Q, Larson DE, Shen D, McLellan MD, Lin L, Miller CA, Mardis ER, Ding L, Wilson RK. VarScan 2: somatic mutation and copy number alteration discovery in cancer by exome sequencing. Genome Res. 2012; 22:568–76. 10.1101/gr.129684.111. 22300766 PMC3290792

[41] Thorvaldsdottir H, Robinson JT, Mesirov JP. Integrative Genomics Viewer (IGV): high-performance genomics data visualization and exploration. Brief Bioinform. 2013; 14:178–92. 10.1093/bib/bbs017. 22517427 PMC3603213

[42] Robinson JT, Thorvaldsdottir H, Winckler W, Guttman M, Lander ES, Getz G, Mesirov JP. Integrative genomics viewer. Nat Biotechnol. 2011; 29:24–6. 10.1038/nbt.1754. 21221095 PMC3346182

[43] Kent WJ. BLAT--the BLAST-like alignment tool. Genome Res. 2002; 12:656–64. 10.1101/gr.229202. 11932250 PMC187518

[44] Kent WJ, Sugnet CW, Furey TS, Roskin KM, Pringle TH, Zahler AM, Haussler D. The human genome browser at UCSC. Genome Res. 2002; 12:996–1006. 10.1101/gr.229102. 12045153 PMC186604

[45] Adzhubei IA, Schmidt S, Peshkin L, Ramensky VE, Gerasimova A, Bork P, Kondrashov AS, Sunyaev SR. A method and server for predicting damaging missense mutations. Nat Methods. 2010; 7:248–9. 10.1038/nmeth0410-248. 20354512 PMC2855889

[46] Schwarz JM, Cooper DN, Schuelke M, Seelow D. MutationTaster2: mutation prediction for the deep-sequencing age. Nat Methods. 2014; 11:361–2. 10.1038/nmeth.2890. 24681721

[47] Reva B, Antipin Y, Sander C. Determinants of protein function revealed by combinatorial entropy optimization. Genome Biol. 2007; 8:R232. 10.1186/gb-2007-8-11-r232. 17976239 PMC2258190

[48] Reva B, Antipin Y, Sander C. Predicting the functional impact of protein mutations: application to cancer genomics. Nucleic Acids Res. 2011; 39:e118. 10.1093/nar/gkr407. 21727090 PMC3177186

[49] Choi Y, Sims GE, Murphy S, Miller JR, Chan AP. Predicting the functional effect of amino acid substitutions and indels. PLoS One. 2012; 7:e46688. 10.1371/journal.pone.0046688. 23056405 PMC3466303

[50] Choi Y. A fast computation of pairwise sequence alignment scores between a protein and a set of single-locus variants of another protein. Proceedings of the ACM Conference on Bioinformatics, Computational Biology and Biomedicine (BCB ’12). 2012; 414–7. 10.1145/2382936.2382989. Available from: http://provean.jcvi.org/downloads/provean.acmbcb.pdf

[51] Kumar P, Henikoff S, Ng PC. Predicting the effects of coding non-synonymous variants on protein function using the SIFT algorithm. Nat Protoc. 2009; 4:1073–81. 10.1038/nprot.2009.86. 19561590

[52] Koressaar T, Remm M. Enhancements and modifications of primer design program Primer3. Bioinformatics. 2007; 23:1289–91. 10.1093/bioinformatics/btm091. 17379693

[53] Untergasser A, Cutcutache I, Koressaar T, Ye J, Faircloth BC, Remm M, Rozen SG. Primer3 - new capabilities and interfaces. Nucleic Acids Res. 2012; 40:e115. 10.1093/nar/gks596. 22730293 PMC3424584

