# Supplementary material for: Deep sequencing of a recurrent oligodendroglioma and the derived xenografts reveals new insights into the evolution of human oligodendroglioma and candidate driver genes
Source: Oncotarget. 2019 Jun 4;10(38):3641–53. doi: 10.18632/oncotarget.26950 (PMC6557204; doi:10.18632/oncotarget.26950)
Supplement: Supplementary file 1 [file oncotarget-10-3641-s001.pdf]

Deep sequencing of a recurrent oligodendroglioma and the derived xenografts reveals new insights into the evolution of human oligodendroglioma and candidate driver genes

SUPPLEMENTARY MATERIALS

Data analysis and biological evaluation

After primary bioinformatics data analysis and a "cleaning process" to differentiate between mouse reads (which are present due to contamination) and xenograft tumor reads (performed by Campos Valenzuela and Kaderali), a statistical and biological filtering as well as a manual verification was performed. We focused only on somatic mutations. We decided to filter for a "normal variant" (occurrence of a mutation in blood) at < 5% and a somatic *p*-value < 0.001 (possibility that mutations occurred randomly is < 0.001%). This analysis yielded 421 somatic SNVs in recurrent O^{2010} and 419 somatic SNVs in xenograft[1]. We then filtered for effects of the mutations. We included non-synonymous-exchange; start gained; start lost; stop gained; stop lost mutations (and for InDels: frame-shift, codon-deletion, codon-change, and codon-gain) in our further studies.

The manual verification includes three steps: a. inspection using the Integrative Genomics Viewer – IGV from the Broadinstitute [1]; b.) applying the BLAT – BLAST-like Alignment Tool [2] and c.) inclusion of information from the UCSC Genome Browser [3]. With the inspection of the sequencing data using IGV, we checked the mapping and base quality and answered the following questions: How many of how many reads showed the called variant? Showed the respective reads bearing the called SNV additional variations and how many additional variants? If reads showed too many changes, there was the assumption that these reads were errors arising during the sequencing process. These reads were not considered for further analysis. In addition, we looked for the changes in the sequencing data obtained from blood. If so, we concluded, that these reads were not error-prone. We checked the positions at which a mutation appeared within a read. If they always appeared in the first or the last few bases of the reads, then we assumed that these mutations were more likely to be false. In the following BLAT-search, we checked the regions bearing the called variants and also included other (in the reads found) changes and mapped them against the human genome (hg19). In the case of variants found in the xenograft, we also blasted against the mouse genome. We checked if the called variants are known ones and if the variants were located in a self chain region or a repeat region. We also checked for other genes at this position and for pseudogenes. We then made the decision as to whether the found variants were true positives/false positives which occur due to contamination with mouse tissue or errors.

In the next step, we applied a biological scoring to understand the impact of these mutations on the protein function. We used Polyphen [4], Mutation taster [5], Mutation assessor [6, 7], SIFT [8–12] and Provean Software [13–15]. If a mutation was deemed benign by all the prediction algorithms, it was given a score of zero and excluded from further analysis. If the mutation was deemed protein damaging by the majority of prediction algorithms, it was given a score of 1. The rest of the mutations were given a score depending on the mutation frequency (1 if < 0.1, 2 if between 0.1 and 0.99, and 3 if > 1). The Variants also received a score of 3 if it was an oncogene or tumor suppressor gene. The number of significant pathways the gene was involved in was also used as a score. A further 1 was given, if it was a transcription factor. All the scores were added up to give an integer falling between 1 and 7. Pathway enrichment and gene attributes were determined using GSEA [16, 17].

REFERENCES

1. Thorvaldsdottir H, Robinson JT, Mesirov JP. Integrative Genomics Viewer (IGV): high-performance genomics data visualization and exploration. Brief Bioinform. 2013; 14:178–192. https://doi.org/10.1093/bib/bbs017. [PubMed]
2. Kent WJ. BLAT--the BLAST-like alignment tool. Genome Res. 2002; 12:656–664. https://doi.org/10.1101/gr.229202. [PubMed]
3. Kent WJ, Sugnet CW, Furey TS, Roskin KM, Pringle TH, Zahler AM, Haussler D. The human genome browser at UCSC. Genome Res. 2002; 12:996–1006. https://doi.org/10.1101/gr.229102. [PubMed]
4. Adzhubei IA, Schmidt S, Peshkin L, Ramensky VE, Gerasimova A, Bork P, Kondrashov AS, Sunyaev SR. A method and server for predicting damaging missense mutations. Nat Methods. 2010; 7:248–249. https://doi.org/10.1038/nmeth0410-248. [PubMed]
5. Schwarz JM, Cooper DN, Schuelke M, Seelow D. MutationTaster2: mutation prediction for the deep-sequencing age. Nat Methods. 2014; 11:361–362. https://doi.org/10.1038/nmeth.2890. [PubMed]

6. Reva B, Antipin Y, Sander C. Determinants of protein function revealed by combinatorial entropy optimization. Genome Biol. 2007; 8:R232. https://doi.org/10.1186/gb-2007-8-11-r232. [PubMed]
7. Reva B, Antipin Y, Sander C. Predicting the functional impact of protein mutations: application to cancer genomics. Nucleic Acids Res. 2011; 39:e118. https://doi.org/10.1093/nar/gkr407. [PubMed]
8. Kumar P, Henikoff S, Ng PC. Predicting the effects of coding non-synonymous variants on protein function using the SIFT algorithm. Nat Protoc. 2009; 4:1073–1081. https://doi.org/10.1038/nprot.2009.86. [PubMed]
9. Ng PC, Henikoff S. Predicting the effects of amino acid substitutions on protein function. Annu Rev Genomics Hum Genet. 2006; 7:61–80. https://doi.org/10.1146/annurev.genom.7.080505.115630. [PubMed]
10. Ng PC, Henikoff S. SIFT: predicting amino acid changes that affect protein function. Nucleic Acids Res. 2003; 31:3812–4. https://doi.org/10.1093/nar/gkg509. [PubMed]
11. Ng PC, Henikoff S. Accounting for Human Polymorphisms Predicted to Affect Protein Function. Genome Res. 2002; 12:436–46. https://doi.org/10.1101/gr.212802. [PubMed]
12. Ng PC, Henikoff S. Predicting Deleterious Amino Acid Substitutions. Genome Res. 2001; 11:863–74. https://doi.org/10.1101/gr.176601. [PubMed]
13. Choi Y, Sims GE, Murphy S, Miller JR, Chan AP. Predicting the functional effect of amino acid substitutions and indels. PLoS One. 2012; 7:e46688. https://doi.org/10.1371/journal.pone.0046688. [PubMed]
14. Choi Y. A Fast Computation of Pairwise Sequence Alignment Scores Between a Protein and a Set of Single-Locus Variants of Another Protein. In Proceedings of the ACM Conference on Bioinformatics, Computational Biology and Biomedicine (BCB '12). 2012; 414–417. https://doi.org/10.1145/2382936.2382989.
15. Choi Y, Chan AP. PROVEAN web server: a tool to predict the functional effect of amino acid substitutions and indels. Bioinformatics. 2015; 31:2745–2747. https://doi.org/10.1093/bioinformatics/btv195. [PubMed]
16. Subramanian A, Tamayo P, Mootha MK, Mukherjee S, Ebert BL, Gillette MA, Paulovich A, Pomeroy SL, Golub TR, Lander ES, Mesirov JP. Gene set enrichment analysis: A knowledge-based approach for interpreting genome-wide expression profiles. PNAS. 2005; 102:15545–15550. https://doi.org/10.1073/pnas.0506580102. [PubMed]
17. Mootha VK, Lindgren CM, Eriksson KF, Subramanian A, Sihag S, Lehar J, Puigserver P, Carlsson E, Ridderstråle M, Laurila E, Houstis N, Daly MJ, Patterson N, et al. GC-1α-responsive genes involved in oxidative phosphorylation are coordinately downregulated in human diabetes. Nature Genetics. 2003; 34:267–273. https://doi.org/10.1038/ng1180. [PubMed]

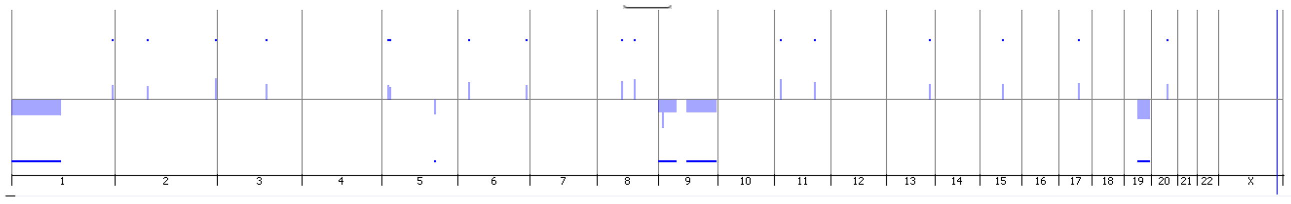

Supplementary Figure 1: aCGHA of primay O^{2005} shows a 1p/19q codeletion.

Supplementary Table 1: Primerlist and PCR conditions for ultra deep sequencing of amplicons

Primer_Name	Primer_F	Primer_R	Additives	Temp in °C
ZNF268	TTCATGGATGTGTTTGTGGATT	ACGGGAAAGATTTGGTTTGG	Q-Solution	63.5
TJP3_2	ATGGAGGATCGTGGGTATGTA	GCAGAATCTGATCTCCCTCCT	/	66.5
MYBL_2	GTGAAGCAGAAGTGCCTATGTC	GGCATGAGGACAATCAGAGG	/	63.5
HECW2_2	GAGTTAGATCTCTGCAGCACCTG	CATACGTGAAGGGTGAGCACT	Formamide	63.5
ARHGAP6_R152P	GTGCCCAGTGCACATTCTT	AATGCCCTTATCCCAAGTCA	DMSO+Betaine	66.5
HERC4 V568A	GACCAGAGCTCCAAAGGACA	TGGTCAGTACTTGAACCTCCA	/	61.5
AXDND1 R883*	CACACTCCCCATCCTACCAG	GAATCCCTGGTCCCCTTAGA	/	63.5
RASAL1	GGACTCAAGGCACACAGGAC	TAGGGTTCTCAGGGCACAAG	Q-Solution	66.5
CCDC70 W164*	GGACAATGCCTTATGGGAAA	GCTCACCCTGGACTTGAATC	/	63.5
ORC1_S819C	CTCGTCTTTCAGCGCATACA	CCAACTGATGCCTTCTGGAG	/	63.5
MAP3K4_E728K	CCCACTTTACCCTGCTGATG	ACTTGGAAGAGGGTGCACAT	Formamide	63.5
TET1_3	ACCCTTGACACTGCATACCC	TAGCAATTGGACACCCATGA	/	63.5
SYPL2A131G	ATGACCCCTTTCTTGGCTCT	CCTCTGCTCCAGTTCTGACC	/	63.5
VSIG2_2	ATTGCTCCCTGCCCTCTACT	AGAGTGGACAAACCTCTGTGG	DMSO & Betaine	66.5
SEMA4A	CCAAGCATCCCTTCTCACAT	TCACAGCGTGACTGGAGAAA	DMSO & Betaine	64.5
TRIM3_2	GAGAGGCAAGATCAGGCAGA	CTATCCTCCTCCAAAGCCCTA	DMSO & Betaine	66.5
PADI1R449Q	GGTGGCAGATCTGATGATGA	ACCCTGGAAGTGTGAGGATG	/	63.5
SYTL2 K92T	TCCCTTCCTTCTCCTCTCTTG	TGATCAATTCCACTAAAACCCTTT	/	66.5
CACNA1S V1461I	CAGAAACAGCCTCTGGGAGA	CCTGATGGAGTGAAGCATTG	/	63.5
NUAK1_3	TTGCTTCGCTTCTTCAGGAT	ATGCTCGAGGACTCATACGG	/	63.5
ATXN2 P731L	GGTATTTGACACCCAGGAATG	CCAGGTCTCCCAGACAGAAC	/	63.5
LUZP1_2	ACTCCTGCTGAAGGCCTCTT	ACATGGCCGAATTTACAAGC	Formamide	64.5
ZC3H10	GACCTACCAAATGGCAAGGA	CAGGTTGCTGACCTGCTTCT	Q-Solution	64
SACS_2	TCATTGGGTCCATAAGCAGA	TGCAAAGCCCTAAGAGAAGG	/	63.5
USPL1_2	TGACGAAACCTCGTCAAACTT	TTGTAAGCAACCGCCAGAAT	/	63.5
ATP7B	CATTCACTGATATCCTCCCTCA	CTGGGTCTGTGGGATTCTTG	/	63.5
MYO5C_2	CGGAGATGATCAGCACAGAA	CACAATATTTCTGGCACCTTTG	/	63.5
PRTG_3	GCAGGGTCTTCACACTTCCT	ATGTGCTCCCACCTGTCTGT	Formamide	63.5
CNOT 1R212K	GCTTTTGTGATGTGTCTGTGG	TTAACCCATTGTGGTGCAAA	/	63.5
PSKH1 R217Q	GAGCGGGTGTACATGGTGAT	TGGTGCCACTGAGTAGGATG	/	63.5
IRF8T267M	CAGGAGGGACCCTGTATGTC	AGACCTGGACCACCTCATCA	DMSO & Betaine	64.5
ZC3H18_2	CTGTCCTGCACACACATGAAC	GAGATAGAGACGGAGGGAGGA	Formamide	66.5
DNAH9 V3435M	CAGCCAGAAAGGCATTCTTC	GGGAACTTCTGTGCTCAACC	/	63.5
LLGL1_2	CTGCCGTACCCTACCCTTTC	CCGGGCTTAGAACTGAAACTC	/	63.5
SPECC1 E172K	CAACCCCAGGAAATCAGTGT	AATTTGGGGAGTGCTCAAGA	/	63.5
LIG3 G131E	GACAATTCAGCCAGTGGTCA	CCTTCCAGCTCTGTGAGGTC	Q-Solution	63.5
RNFT1 P301S	TGTGAATCATGCAGTGACAAG	TTCTTTCAAAGCAGGCCACT	Q-Solution	64.5
DSC3_3	ACTATCTGGGCAAGGCACAT	GCGTCAACTGCAGATGGATA	/	63.5
FHOD3 L76P	ACAAAACACAGGAGGGCAAC	CGAGCACCCATCAAGAAAAG	/	63.5
DYM T569I	CACACACACAGATTACCACAACT	TTGCAAGGAGGAAAAGACTGA	/	63.5
NWD1_2	AGCACAGGTGGTCAACAGAGT	TCTGTGCGGAAAGGAGAAGTA	/	66.5
CIC_x19	TAGAATGCAGTGAGGGCTTG	TTGGAGGGAAAGATGTCTGC	Formamide	63.5
SLC35F5 G318E	CCTGCTAGTGCCTACCCTTT	TTGCTAAATTATTTGATAGTTTTACCC	/	61.5
USP25 E335K	TTCAGGTTCAGGTGAGAATCC	GGCCATATTGACTGGACAGG	/	63.5

DHRS9_2	GGGTCTGATCAATAATGCTGGT	TTGAAAGGTACTAAATAGGATGTCTTG	/	63.5
ZAK	ATTTGGGCCAACTGTTCAGA	ACTTGAGTCGGACCTGCTTC	/	63.5
NBEAL1_2	CAGGAACACAATTAGCAGTCAGA	CCAGTTCCATGTTATCCCAGTT	/	66.5
IDH1_GlioM_R132H	TGTGTTGAGATGGACGCCTA	TCCTGATGAGAAGAGGGTTGA	/	63.5
CABP7	TCTCAGAGCACCGTGTTGTC	GGACATCAAAGCGATGGAGT	Q-Solution.	63.5
SMCR7L_2	ACCCTTTAAATTCTGCCCTGA	GATGGCAGGAAGTCAATGAAG	/	66.5
TCF20 Q833*	CCAGGAAGAATGACCGACTG	TGGCTGGTAGCCTAGAAGGA	/	63.5
PLEKHH2_2	TTGCTGTAATTCTCACAAAGTGG	ATCCCATTTTCAGCGATCAT	/	63.5
PELI1_2	CTGAACACACATGCCCACA	TGGCTCGTTAATTGACCTCTG	Formamide	66.5
DUSP11_2	GCAAATGAGGTAGCCAGTCC	ATCAGTTGCTGATGGGCATT	/	63.5
C3orf15	TGAAAGTGAATTGACTGCCAAG	TTTGGTTTGACTTGGCTCCT	/	63.5
TGFBR2 E167D	CCTCGCTTCCAATGAATCTC	AAACCCTGCTACTCCCCAAG	Formamide	64.5
COL25A1_2_F,<br>COL25A1G251R_R	CACATCCAAAATACTTGAAATGGA	TGAACACAGTGGATAAGGTCTGA	/	66.5
HS3ST1_2	AGGCAGTAAAAGCCCTTGGT	AGCGAGTCTACAGCATGAACC	/	63.5
NDST4_2	GGAAATGAAATCAAGCATGACA	GAAATGATGCCTATGGCAAAC	/	66.5
ZCCHC4 S158N	AGCCAACATTCCACAGATTTC	GAATCCGAGGGCAGAAAGTA	/	63.5
ZNF608 Y939*	CTGCGTGCTCCCACTATTTC	GAATGCAGCACTTTGGTGAA	/	63.5
SRA1	CCACTGGGTCAGAGGGTCTA	ACCCATTTAAGCCATGTTGC	Formamide	63.5
EPHA1_3	AGACAGGCCTGACAGTGACTC	AGTGTGAGGTGTTCCCAGTGT	Q-Solution.	66.5
GLI3_3	TGTGAAGCAGCAGAACAGAA	CCCTACATTAATCCCTACATGGAC	/	61.5
ABCB4_2	GCCATCAGTAAAGGGTGCTT	GGATCCAGAACACTGGCATT	/	63.5
ODF1_3	GGACAGAGAACTAAGGCAACTGA	TTTCCTAAGTCACCAGGGTCAT	/	66.5
CPSF1_2	CTTGCTTTCCTCCTGGTAGC	TGGATGTGATTGAGGTGGTG	DMSO & Betaine	66.5
EXTL3 E419K	CAGGGCGAGAAGATTGAGTC	CACTGCAGCATGTCCTGGTA	/	63.5
RNF122 Q145*	GCATGGAGTAGCAGGGAAGA	CAGCGTGGTCTGTCTGGTTA	DMSO & Betaine	64.5
RNF122_D99N	ACCTAATCCCTCTGGGCTTT	GTTTCTCTGCCTGGTAGAACAGA	/	63.5
BAAT S31N	CCTTTGGAGCACTGGCAACT	CCTGAAGAATCATCCCAGGT	/	63.5
NAIF1_2	TGCAACCCCTAAGCGTTTATT	CAACCTGCATGTGAAGGAGA	/	63.5
TMEM203 R134Q	GCGGTGCCTTCAGACTAGAG	AACGTGTTCGTGCCTTTCTT	/	63.5
DCAF10 T253I	TCCTGGCTCTGTGATTATGC	TGACATTTCCATCAAATCCTGA	DMSO & Betaine	64.5
PTCHD1	CGCTCTGCTCTAGGATGCTG	GTCCAGCATGTTGGCTTTCT	Formamide	64.5
BCOR A505T	GTTTTGGTGCCATCTGCATT	ATGCTTCCAAAGCTGACCAC	/	63.5
SNX12	TCCTAGCAGAGTGCCCACAT	TGCTGTCCTCTACTGGCTTG	Q-Solution	68
SHROOM2	TGAGAGCAGTCCAGAGGACA	CCCGCCACTTGTAGATGAG	/	63.5
KPNA1_2	CCTCATGAAAGCCTCCATCT	AATGTCATATAGTGTCAGACCTCTCTT	/	61.5
RBPJ_2	TCGACTACGATCCCAGACAGT	AAAGCAAAAGTAGAGCCATACCA	/	66.5
NUP210LC(691)-	CTGAAAAGGAATGGCTGGAA	CAAGTGAGAGGCATGGCATA	Q-Solution	63.5
NPHS2 A(118)-	CAGGAAATTACCTATTGGGTCCT	CCCACTACCTTTCCAGGAG	/	61.5
ARID1A0(764)C	TCCCAGGATAAGGATGGAGA	GGGTGGATTAAAGGCAAAGG	/	63.5
FUBP1 G(436)-	TTGCTGGAAAATCTTCACGTT	GGCCCATTTAATTGTGACCA	/	63.5
SMAD7_3	AAACGAGGACGAGAAGAAGAA	GTGGATGGTGTGTGGGTGTA	Formamide	63.5
VILL_2	TGACAGCCACAACACCAG	GCTCTGACCTGCGGACTCT	Q-Solution	65,5
TBP CAA(72)-	TTCTCCTTGCTTTCCACAGG	GAGTCATGGGGGAGGGATAC	DMSO & Betaine	64.5

Supplementary Table 2: Primerlist for Sanger validation of variations found in primary and TERT promotor

Primer_Name	Primer_F	Primer_R	Additives	Temp in °C
SYPL_Val	ACCTCATGGGGGACTTCTCT	GTTTCTCCACCCCATTCCTT	/	66
ATP7B_Val	CGAGGACAAATGGCATCAC	GCTATATTTTCTCATTTTTCTTCACTG	/	63.5
PSKH1_Val	ATTGCCAAGGGCTCCTTCA	CGGTGATGATGATCTTGGAG	/	64
DUSP11_Val	TTCATTTTCAGGGCAGTGAT	GGTTGGCAACTTACCTGCAA	/	64
C3orf15_Val	AGACTGCAGGAGGAGAGGAG	TTCTAGGCAGCTTCCACACA	Formamide	66
EPHA1_Val_2	AATGGCCTTGAACCTTATGC	CCATGCTGATGCTGACTGAG	/	60
SNX12_Val	CCATCCTGGTTTCTTTATAGAATTG	AAAAACCAATGTCATTTCAGCA	/	64
NUP210L_Val	ATTTGAAGGGGGTCCTCGT	GCATTGGATCCGGTAGATGT	/	61
SMAD7_Val_3	ACAAGAGTCAGCTGGTGCAG	GAACACCTTGTGTACCAACAGC	/	60
IDH1_GlioM_R132H	TGTGTTGAGATGGACGCCTA	TCCTGATGAGAAGAGGGTTGA	/	63.5
TERT_reg1_1	GGCCGATTCGACCTCTCT	AGCACCTCGCGGTAGTGG	Q-Solution	60

Supplementary Table 3: Variants found in patients Oligodendroglioma and patients derivedxenograft models

Gene	Score	Coding region change	Amino acid change	Effekt	occurance	allele frequency				
						primary O^{2005}	recurrent O^{2010}	xenograf[1]	xenograft[2]	blood
ZNF268	0	NM_003415:c.295G>A	NM_003415:p.Asp99Asn	missense	somatic-recurrent	0	44%	50%	50%	0
TJP3	3	NM_001267560:c.1174A>T	NM_001267560:p.Ile392Phe	missense	somatic-recurrent	0	45%	47%	58%	
MYBL2	4	NM_002466:c.101G>A	NM_002466:p.Trp34*	stop gained	somatic-recurrent	0	45%	49%	39%	
HECW2	4	NM_020760:c.2446C>T	NM_020760:p.Arg816Cys	missense	somatic-recurrent	0	43%	47%	50%	
ARHGAP6	0	NM_013423:c.455G>C	NM_013423:p.Arg152Pro	missense	mosaik in recurrent	0	12%	0	0	
HERC4	6	NM_015601:c.1703T>C	NM_015601:p.Val568Ala	missense	somatic-xenograft	0	0	47%	48%	
AXDND1	6	NM_144696.4:c.2647C>T	NM_144696.4:p.Arg883*	stop gained	somatic-xenograft	0	0	28%	31%	
RASAL1	0	NM_001193520:c.2305C>T	NM_001193520:p.Arg769Trp	missense	somatic-recurrent	0	43%	46%	52%	
CCDC70	4	NM_031290.2:c.491G>A	NM_031290.2:p.Trp164*	stop_gained	somatic-xenograft	0	0	31%	34%	
ORC1	0	NM_001190819.1:c.2456C>G	NM_001190819.1:p.Ser819Cys	missense	somatic-xenograft	0	0	100%	99%	
MAP3K4	6	NM_005922.2:c.2182G>A	NM_005922.2:p.Glu728Lys	missense	somatic-xenograft	0	0	26%	27%	
TET1	2	NM_030625:c.4399G>A	NM_030625:p.Glu1467Lys	missense	somatic-recurrent	0	43%	51%	47%	
SYPL2	2	NM_001040709:c.392C>G	NM_001040709:p.Ala131Gly	missense	somatic-primary	20%	84%	100%	100%	0
VSIG2	2	NM_014312.3:c.506C>T	NM_014312.3:p.Ser169Phe	missense	somatic-recurrent	0	44%	64%	62%	
SEMA4A	3	NM_022367.3:c.511G>A	NM_022367.3:p.Glu171Lys	missense	somatic-recurrent	0	45%	48%	47%	
TRIM3	2	NM_006458:c.2020G>A	NM_006458:p.Gly674Arg	missense	somatic-recurrent	0	81%	100%	100%	0
PADI1	2	NM_013358:c.1346G>A	NM_013358:p.Arg449Gln	missense	somatic-recurrent	0	83%	100%	99%	
SYTL2	3	NM_032943.2:c.1997A>C	NM_032943.2:p.Lys666Thr	missense	somatic-recurrent	0	43%	33%	27%	
CACNA1S	2	NM_000069.2:c.4381G>A	NM_000069.2:p.[al1461Ile	missense	somatic-recurrent	0	45%	49%	55%	
NUAK1	3	NM_014840:c.1185G>T	NM_014840:p.Lys395Asn	missense	somatic-recurrent	0	91%	100%	99%	
ATXN2	3	NM_002973:c.2192C>T	NM_002973:p.[Pro731Leu	missense	somatic-recurrent	0	45%	45%	51%	
LUZP1	1	NM_033631:c.214G>A	NM_033631:p.Val72Met	missense	somatic-recurrent	0	87%	100%	100%	
ZC3H10	2	NM_032786:c.509G>A	NM_032786:p.Gly170Glu	missense	somatic-recurrent	0	44%	48%	43%	
SACS	2	NM_014363:c.12302C>T	NM_014363:p.Ala4101Val	missense	somatic-recurrent	0	41%	47%	51%	
USPL1	2	NM_005800:c.715G>A	NM_005800:p.Asp239Asn	missense	somatic-recurrent	0	43%	45%	45%	
ATP7B	3	NM_000053:c.1829C>T	NM_000053:p.Pro610Leu	missense	somatic-primary	3%	45%	48%	47%	0
MYO5C	4	NM_018728:c.2341C>T	NM_018728:p.Arg781*	missense	somatic-recurrent	0	45%	50%	50%	
PRTG	1	NM_173814:c.3334G>A	NM_173814:p.Glu1112Lys	missense	somatic-recurrent	0	44%	50	51%	
CNOT1	1	NM_016284:c.635G>A	NM_016284:p.Arg212Lys	missense	somatic-recurrent	0	45%	46%	54%	
PSKH1	4	NM_006742:c.650G>A	NM_006742:p.Arg217Gln	missense	somatic-primary	63%	44%	48%	52%	0
IRF8	4	NM_002163:c.800C>T	NM_002163:p.Thr267Met	missense	somatic-recurrent	0	46%	49%	50%	
ZC3H18	2	NM_144604:c.1808C>G	NM_144604:p.Ser603Cys	missense	somatic-recurrent	0	41%	48%	47%	
DNAH9	2	NM_001372:c.10303G>A	NM_001372:p.Val3435Met	missense	somatic-recurrent	0	42%	48%	47%	
LLGL1	2	NM_004140:c.1210G>A	NM_004140:p.Val404Ile	missense	somatic-recurrent	0	43%	49%	51%	
SPECC1	6	NM_152904:c.514G>A	NM_152904:p.Glu172Lys	missense	somatic-recurrent	0	43%	49%	45%	
LIG3	2	NM_002311:c.392G>A	NM_002311:p.Gly131Glu	missense	somatic-recurrent	0	44%	48%	42%	
RNFT1	2	NM_016125:c.901C>T	NM_016125:p.Pro301Ser	missense	somatic-recurrent	0	44%	50%	52%	
DSC3	2	NM_001941:c.731C>G	NM_001941:p.Thr244Arg	missense	somatic-recurrent	0	43%	50%	49%	
FHOD3	2	NM_025135:c.227T>C	NM_025135:p.Leu76Pro	missense	somatic-recurrent	0	42%	48%	50%	
DYM	2	NM_017653:c.1706C>T	NM_017653:p.Thr569Ile	missense	somatic-recurrent	0	45%	46%	50%	
NWD1	2	NM_001007525:c.2074G>A	NM_001007525:p.Gly692Ser	missense	somatic-recurrent	0	45%	48%	49%	
CIC	5	NM_015125:c.4421T>G	NM_015125:p.Val1474Gly	missense	somatic-recurrent	0	88%	100%	100%	
SLC35F5	2	NM_025181:c.953G>A	NM_025181:p.Gly318Glu	missense	somatic-recurrent	0	45%	45%	47%	
USP25	1	NM_013396:c.1003G>A	NM_013396:p.Glu335Lys	missense	somatic-recurrent	0	46%	50%	48%	
DHRS9	0	NM_005771:c.428G>A	NM_005771:p.Ser143Asn	missense	somatic-recurrent	0	40%	28%	50%	

ZAK	5	NM_016653:c.1618C>T	NM_016653:p.Gln540*	stop gained	somatic-recurrent	0	22%	48%	53%	
NBEAL1	3	NM_001114132:c.5554C>T	NM_001114132:p.Gln1852*	stop gained	somatic-recurrent	0	43%	45%	49%	
IDH1	6	NM_005896:c.395G>A	NM_005896:p.Arg132His	missense	somatic-primary	37%	43%	49%	46%	0
CABP7	2	NM_182527:c.465G>A	NM_182527:p.Met155Ile	missense	somatic-recurrent	0	47%	47%	49%	
SMCR7L	4	NM_019008:c.589G>A	NM_019008:p.Val197Met	missense	somatic-recurrent	0	45%	48%	53%	
TCF20	4	NM_005650:c.2497C>T	NM_005650:p.Gln833*	stop gained	somatic-recurrent	0	41%	49%	50%	
PLEKHH2	2	NM_172069:c.2446G>A	NM_172069:p.Gly816Arg	missense	somatic-recurrent	0	45%	51%	54%	
PELI1	4	NM_020651:c.835C>T	NM_020651:p.Arg279*	stop gained	somatic-recurrent	0	45%	47%	48%	
DUSP11	2	NM_003584:c.575A>G	NM_003584:p.Asp192Gly	missense	somatic-primary	20%	46%	51%	50%	0
C3orf15	3	NM_033364:c.1820G>A	NM_033364:p.Arg607Gln	missense	somatic-primary	11%	45%	51%	40%	0
TGFBR2	3	NM_003242:c.426G>C	NM_003242:p.Glu142Asp	missense	somatic-recurrent	0	42%	50%	45%	
COL25A1	3	NM_032518:c.763G>C	NM_032518:p.Gly255Arg	missense	somatic-recurrent	0	42%	50%	45%	
HS3ST1	4	NM_005114:c.656G>A	NM_005114:p.Arg219His	missense	somatic-recurrent	0	76%	94%	76%	
NDST4	4	NM_022569:c.2392C>G	NM_022569:p.Leu798Val	missense	somatic-primary	18%	84%	100%	100%	0
ZCCHC4	2	NM_024936:c.473G>A	NM_024936:p.Ser158Asn	missense	somatic-recurrent	0	85%	100%	100%	
ZNF608	3	NM_020747:c.2817T>G	NM_020747:p.Tyr939*	stop gained	somatic-recurrent	0	47%	50%	48%	
SRA1	4	NM_001035235:c.262C>T	NM_001035235:p.Pro88Ser	missense	somatic-recurrent	0	47%	50%	52%	
EPHA1	3	NM_005232:c.1262C>A	NM_005232:p.Ala421Asp	missense	somatic-primary	10%	45%	47%	43%	0
GLI3	3	NM_000168:c.676G>T	NM_000168:p.Asp226Tyr	missense	somatic-recurrent	0	46%	47%	52%	
ABCB4	2	NM_000443:c.811G>A	NM_000443:p.Gly271Ser	missense	somatic-recurrent	0	47%	50%	56%	
ODF1	3	NM_024410:c.298G>A	NM_024410:p.Asp100Asn	missense	somatic-recurrent	0	42%	48%	47%	
CPSF1	3	NM_013291:c.3461C>T	NM_013291:p.Pro1154Leu	missense	somatic-recurrent	0	44%	50%	49%	
EXTL3	2	NM_001440:c.1255G>A	NM_001440:p.Glu419Lys	missense	somatic-recurrent	0	43%	43%	37%	
RNF122	6	NM_024787:c.433C>T	NM_024787:p.Gln145*	stop gained	somatic-recurrent	0	46%	50%	55%	
RNF122	5	NM_024787:c.294_295delAGinsGA	NM_024787:p.Asp99Asn	missense	somatic-recurrent	0	42%	45%	45%	
BAAT	5	NM_001701:c.92G>A	NM_001701:p.Ser31Asn	missense	somatic-recurrent	0	44%	45%	47%	
NAIF1	3	NM_197956:c.958G>A	NM_197956:p.Gly320Arg	missense	somatic-recurrent	0	44%	47%	48%	
TMEM203	2	NM_053045.1:c.401G>A	NM_053045.1:p.Arg134Gln	missense	somatic-recurrent	0	45%	48%	50%	
DCAF10	2	NM_024345:c.758C>T	NM_024345:p.Thr253Ile	missense	somatic-recurrent	0	25%	28%	27%	
PTCHD1	2	NM_173495:c.53G>C	NM_173495:p.Gly18Ala	missense	somatic-recurrent	0	32%	37%	27%	
BCOR	1	NM_017745:c.1513G>A	NM_017745:p.Ala505Thr	missense	somatic-recurrent	0	42%	47%	45%	
SNX12	3	NM_001256188:c.470G>A	NM_001256188:p.Arg157His	missense	somatic-primary	56%	44%	49%	46%	0
SHROOM2	0	NM_001649:c.1942G>T	NM_001649:p.Val648Leu	missense	somtic-recurrent	0	26%	47%	39%	
SHROOM2		NM_001649.2:c.1936_40delAGCAC	NM_001649.2:p.Thr647Glyfs*33	frameshift	somtic-recurrent	0	19%	34%	31%	
KPNA1		NM_002264:c.179_181delAAG	NM_002264:p.lu60del	codon change plus codon deletion	somatic-recurrent	0	20%	45%	43%	
RBPJ		NM_015874:c.706_707delAA	NM_015874:p.Lys236fs	frameshift	somatic-xenograft			95%	95%	
NUP210L		NM_207308:c.2072delT	NM_207308:p.Leu691fs	frameshift	germline?	1.2%	37%	41%	36%	1.1%
NPHS2		NM_014625:c.353delC	NM_014625:p.Pro118fs	frameshift	somatic-recurrent	0	43%	50%	49%	
ARID1A		NM_006015:c. 2290_2291insC	NM_006015:p.Gln766fs	frameshift	somatic-recurrent	0	75%	89%	89%	
FUBP1		NM_003902:c.1307_1310delTAGA	NM_003902:p.Ile436fs	frameshift	somatic-recurrent	0	61%	83%	79%	
SMAD7		NM_001190823:c.484_485insT	NM_001190823:p.Pro162fs	frameshift	somatic-primary	8%	49%	49%	46%	0
VILL		NM_015873:c.1648_1652delTGGTT	NM_015873:p.Phe551fs	frameshift	somatic-recurrent	0	18%	24%	22%	

Supplementary Table 4: Mutations and their validation by different prediction algorithms found in primary O^{2005}

Gene name	chromosomal region		effect	associated Diseases from OMIM, KEGG, COSMIC	One or more variants in this gene is in a regulatory motif	SIFT Score, (Median Info)	Mutation Assessor (l = low; m = medium; h = high)	Mutation Taster	PolyPhen 2	PROVEAN	ExaC
SYPL2	1p13.3	NM_001040709.1:c.[392C>G	Missense			tolerated 0.11(2.73)	l	disease causing 0.77	Restriction sites created and deleted; transmembrane: affected; probably damaging 0.999	deleterious	not in
PSKH1	16q22.1	NM_006742.2:c.[650G>A	Missense	Y COSM 972616	Y	damaging: 0.00(3.05)	h:3.795	disease causing 0.999	Restriction sites deleted; nuclear; probably damaging 0.999	deleterious	not in
DUSP11	2p13.1	NM_003584.2:c.[575A>G	Missense			tolerated 0.61(2.78)	l	disease causing 0.995	Ubiquitylation; Benign	deleterious	not in
C3orf15	3q13.33	NM_033364.3:c.[1820G>A	Missense			damaging 0.00(2.87)	m	disease causing 0.918	probably damaging 1.000	deleterious	not in
SNX12	Xq13.1	NM_001256188:c.[470G>A	Missense	N	Y	damaging <Warning! Low confidence>	m: 3.015	disease causing 0.999	Human Var: damaging:0.937; Human Div: damaging:0.999	deleterious	not in
SMAD7	18q21.1	NM_001190823:c.[484_485insT	Frame shift					disease causing 1			not in
NUP210L	1q21.3	NM_207308:c.[2072delT	Frame shift					disease causing 1			CA/C Allele frequency 0.00004191
ATP7B	13q14.3	NM_000053:c.[1829C>T	Missense			tolerated 0.07(2,78)	m	disease Causing 0.586	possibly damaging 0.935	deleterious	C>T; Allele frequency: 0.00003312;
EPHA1	7q34	NM_005232.4:c.[1262C>A	Missense	Y OMIM	Y		m	disease causing 0.9567	possibly damaging 0.996	deleterious	C>T; Allele frequency 0.00001651
NDST4	4q26	NM_022569.1:c.[2392C>G	Missense			damaging 0.01 (2.76)	m	disease causing 0.999	possibly damaging 0.749	neutral	not in
